# A history of the MetaSUB consortium: Tracking urban microbes around the globe

**DOI:** 10.1016/j.isci.2022.104993

**Published:** 2022-10-20

**Authors:** Krista A. Ryon, Braden T. Tierney, Alina Frolova, Andre Kahles, Christelle Desnues, Christos Ouzounis, Cynthis Gibas, Daniela Bezdan, Youping Deng, Ding He, Emmanuel Dias-Neto, Eran Elhaik, Evan Afshin, George Grills, Gregorio Iraola, Haruo Suzuki, Johannes Werner, Klas Udekwu, Lynn Schriml, Malay Bhattacharyya, Manuela Oliveira, Maria Mercedes Zambrano, Nur Hazlin Hazrin-Chong, Olayinka Osuolale, Paweł P. Łabaj, Prisca Tiasse, Sampath Rapuri, Silvia Borras, Sofya Pozdniakova, Tieliu Shi, Ugur Sezerman, Xavier Rodo, Zehra Hazal Sezer, Christopher E. Mason

**Affiliations:** 1 Institute of Molecular Biology and Genetics of NASU, Kyiv, Ukraine; 2 Kyiv Academic University, Kyiv, Ukraine; 3 ETH Zurich, Zurich, Switzerland; 4 Department of Physiology and Biophysics, Weill Cornell Medicine, New York, NY USA; 5 Weill Cornell Medicine, New York, NY, USA; 6 Mediterranean Institute of Oceanography, 163 Avenue de Luminy Bâtiment Méditerranée, 13288, Marseille Cedex 9, France; 7 Department of Physiology and Biophysics, Weill Cornell Medicine, New York, NY, USA; 8 WorldQuant Initiative for Quantitative Prediction, New York, NY, USA; 9 Aristotle University of Thessalonica & CERTH, Thessaloniki, Greece; 10 University of North Carolina, Charlotte, NC, USA; 11 Institute of Medical Genetics and Applied Genomics, University of Tübingen, Tübingen, Germany; 12 GermanyNGS Competence Center Tübingen (NCCT), University of Tübingen, Tübingen, Germany; 13 yuri GmbH, Meckenbeuren, Germany; 14 University of Copenhagen, Copenhagen, Denmark; 15 AC Camargo Cancer Center, Sao Paulo, Brazil; 16 Lund University, Lund, Sweden; 17 University of Miami, Miami, FL, USA; 18 Institut Pasteur de Montevideo, Mataojo 2020, Montevideo, 11400, Uruguay; 19 Keio University, Tokyo, Japan; 20 High Performance and Cloud Computing Group, Zentrum für Datenverarbeitung (ZDV), Eberhard Karls University of Tübingen, Tübingen, Germany; 21 Department of Medical Sciences, Uppsala University, Uppsala, Sweden; 22 University of Maryland School of Medicine, University of Maryland, Baltimore, MD, USA; 23 Indian Statistical Institute, Kolkata, India; 24 University of Porto, Porto, Portugal; 25 Corporación Corpogen, Bogotá, Columbia; 26 Universiti Kebangsaan Malaysia, Bangi, Malaysia; 27 Elizade University, Ilara-Mokin, Nigeria; 28 MCB Jagiellonian University, Kraków, Poland; 29 MCB Jagiellonian University, Kraków, Poland; 30 The Community Lab; Los Alamos Makers, Los Alamos, NM 87544, USA; 31 Barcelona Institute for Global Health, Rosselló, 132, 708036 Barcelona, Spain; 32 East China Normal University, Zhongshan Rd (N), 3663, 200050 Shanghai, Putuo, China; 33 Department of Biostatistics, Acibadem University, Istanbul, Turkey; 34 University of Tübingen, Tübingen, Germany; 35 Swiss Institute of Bioinformatics, Lausanne, Switzerland; 36 Department of Quantitative Health Sciences, John A. Burns School of Medicine, University of Hawaii at Manoa, Honolulu, HI 96813, USA

## Abstract

The MetaSUB Consortium, founded in 2015, is a global consortium with an interdisciplinary team of clinicians, scientists, bioinformaticians, engineers, and designers, with members from more than 100 countries across the globe. This network has continually collected samples from urban and rural sites including subways and transit systems, sewage systems, hospitals, and other environmental sampling. These collections have been ongoing since 2015 and have continued when possible, even throughout the COVID-19 pandemic. The consortium has optimized their workflow for the collection, isolation, and sequencing of DNA and RNA collected from these various sites and processing them for metagenomics analysis, including the identification of SARS-CoV-2 and its variants. Here, the Consortium describes its foundations, and its ongoing work to expand on this network and to focus its scope on the mapping, annotation, and prediction of emerging pathogens, mapping microbial evolution and antibiotic resistance, and the discovery of novel organisms and biosynthetic gene clusters.

**Figure:**
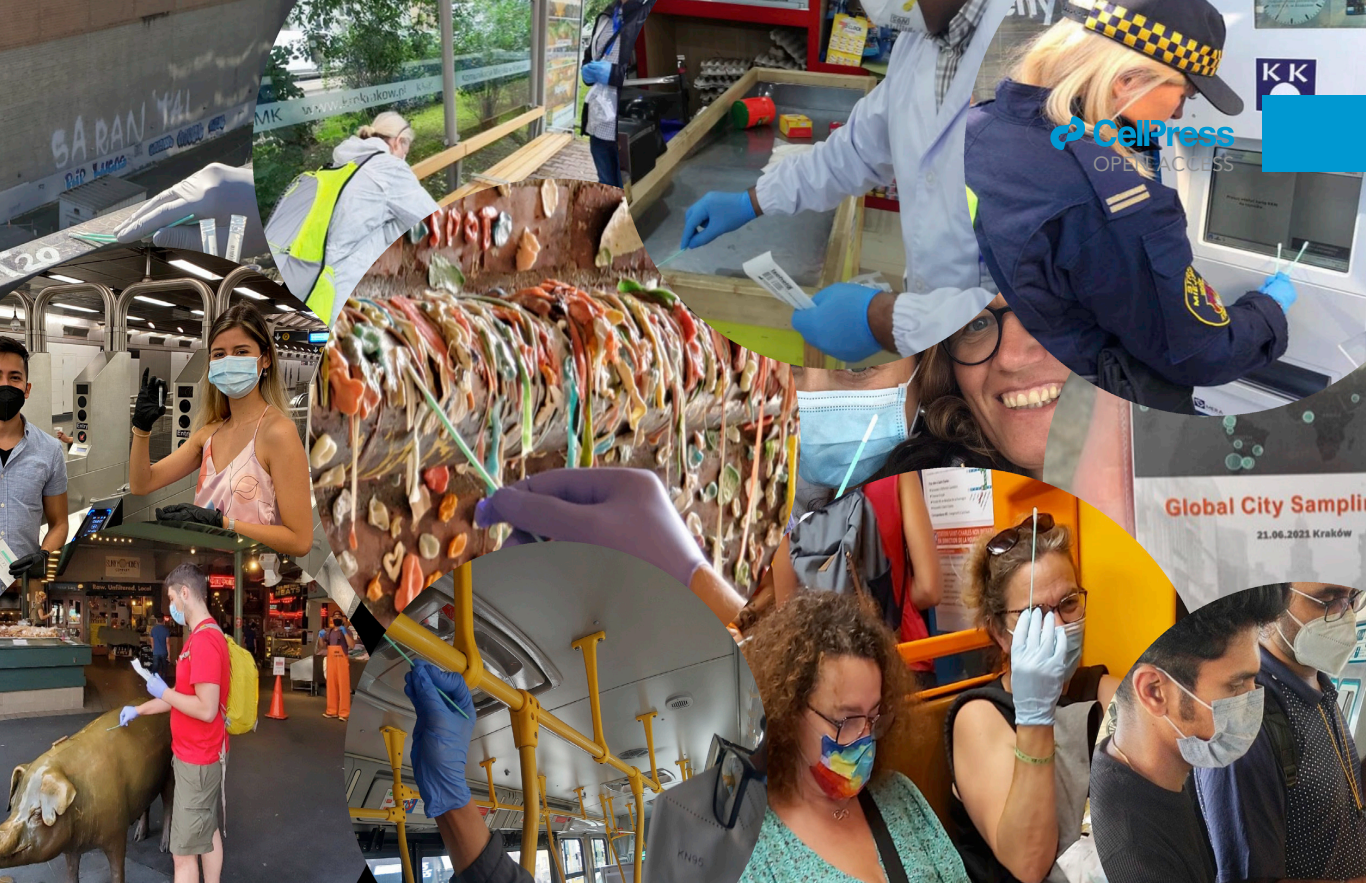
Collage of participants of the MetaSUB consortium

“The collaborations for this project are fortuitous: sometimes it is people reaching out because they want to look at their city relative to another, and sometimes it is just a simple burst of curiosity.”

“We continue to improve logistics, solve challenges, and track down microbial mysteries, but the fantastic group of people in MetaSUB discusses these problems openly to find a solution. For a consortium that is so diverse, we work well together.”

“… this work opened avenues for future research (e.g. novel CRISPR arrays, new species of microbes, and new peptides) that will educate and enable scientists, policymakers, and public health officials to identify infectious microbial populations, assess risks, map outbreaks, and devise strategies to manage and mitigate the epidemic threats.”

“Comparison with global datasets revealed a massive expansion of microbial biodiversity associated with the human body. The integration of this data with environmental metagenomes from MetaSUB will enable unprecedented comparisons of the ecological relationships between the human microbiome and its surrounding environment.”

“Overall, what we are doing is a real example of a modern-day team science approach and bringing in colleagues with specialties in diverse fields. We have people from different backgrounds such as microbiology, ecology, bioinformatics, computational genomics, design, engineering, diagnostics, and clinical care.”

“Microbes are miraculous, they evolve much faster than humans, and this is also valid for MetaSUB. It has truly been evolving over the years.”

## Main text

Environmental microbes play a major role in human life, from causing diseases to potentially preventing or curing them. To understand this complex relationship, adding missing components of the studies on human “exposome”, a group of international investigators came together and formed “The International Metagenomics and Metadesign of Subways and Urban Biomes (MetaSUB) consortium” (www.metasub.org) under the leadership of Dr. Christopher Mason of Weill Cornell Medicine. The MetaSUB consortium aims to map the genome of microbes from environmental samples, or the “metagenome” collected from urban environments. It started with swab samples from the New York City subway system, and has now expanded to over 100 cities across continents with a group of curious, enthusiastic, and knowledgeable collaborators.

**Figure:**
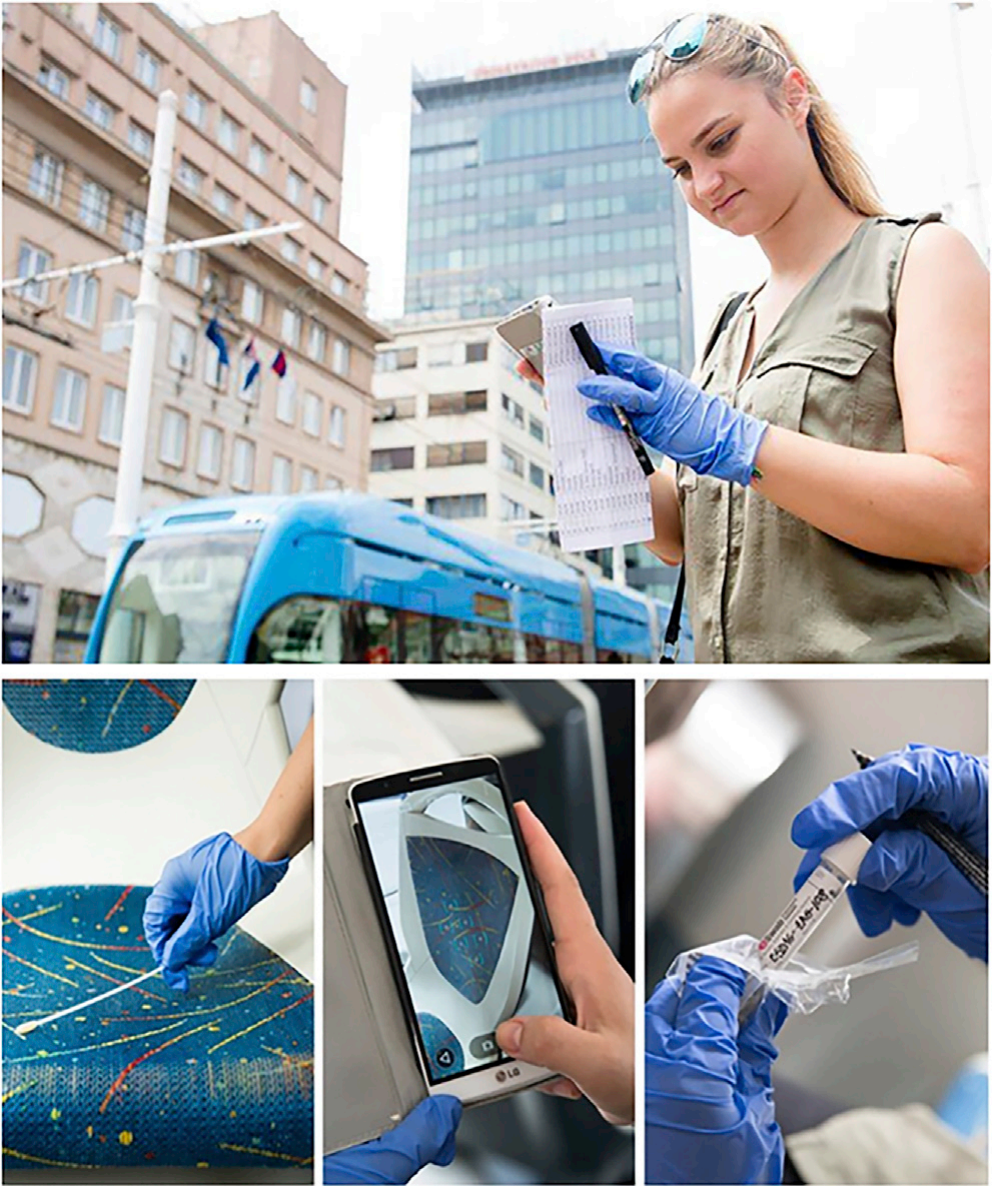
Depiction of the collection process typical of the MetaSUB consortium

The consortium’s work was recently highlighted in *Cell* (Danko et al., 2021a). They collected 4,728 samples from global mass transit systems over three years across 60 urban cities in 32 countries and six continents (PMID: 34043940). Employing shotgun DNA sequencing and creating an open-source MetaSUB Core Analysis Pipeline, the team identified microbial (bacteria, archaea, fungi, protists, and viruses) signatures distinct from human commensal microbes and showed that every city has its own microbiome signature echo. The results also showed an uneven distribution of antimicrobial resistance (AMR) genes in various cities. This global genetic atlas, focused on urban and mass-transit microbiomes, provides new insights into the density, types, and dynamics of antimicrobial resistance (AMR) and the interaction of microbes in the areas of the highest human population density. Furthermore, this work opened avenues for future research (e.g. novel CRISPR arrays, new species of microbes, and new peptides) that will educate and enable scientists, policymakers, and public health officials to identify infectious microbial populations, assess risks, map outbreaks, and devise strategies to manage and mitigate the epidemic threats (Wu et al., 2022).

In this Backstory, the *iScience* editorial team interviewed several members of the MetaSUB consortium to peek into their exciting journey about how this interdisciplinary idea was conceived, how the research collaborations were formed, and how they view the future of such collaborative research that has potential to impact both the scientific community and general population.

### Early days: Swabs and subways

#### How did the MetaSUB consortium begin?

##### Christopher Mason (Weill Cornell Medicine, New York City, USA)

I was traveling on the New York subway with my daughter when she was very young, and she did something that got me thinking—she licked the subway pole.

At first I was alarmed, desperately alarmed, but I then started wondering about the biology of the situation: specifically, what microorganisms were living in our subways? Which ones could transfer from a spontaneous licking incident? Eventually, it dawned on me, and other members of our team, that, with modern sequencing technology, we could quantify and map the microbial diversity of our cities. And where better to start than New York City?

As a result, the MetaSUB consortium, and projects under it, was co-founded by myself and Dr. Evan Afshin. It all began after we published a paper in 2015 in *Cell Systems* reporting a snapshot of the microbial population from the swabs collected from the surfaces across the New York City subway system (Afshinnekoo et al., 2015).

##### Evan Afshin (Weill Cornell Medicine, New York City, USA)

As Chris said, as soon as we began reaching out to different cities and collaborators, the list of cities and people who wanted to join the consortium quickly grew. The collaborations for this project are fortuitous: sometimes it is people reaching out because they want to look at their city relative to another, and sometimes it is just a simple burst of curiosity. We soon had 60 cities that were interested in sampling.

Our first inaugural meeting was at the New York Genome Center in 2015 (reported in The MetaSUB International Consortium et al., 2016). During the meeting, we created working groups and discussed some of the consortium’s main goals and challenges in sample collection and processing, metadata organization and standardization, bioinformatics analyses, and public outreach and engagement.

##### Mason

The collaborations for this project arose organically, and no two came about in the same way. Dr. Emmanuel Dias-Neto joined the consortium after we met following a talk I gave in Brazil. The collaboration with Dr. Patrick Lee was established after our team read their pioneering work on the atmospheric microbiome in ventilated buildings at a city scale in Hong Kong across seasons (Zhou et al., 2020). In 2016, we focused on building the consortium, and as a result, we were able to connect with people around the world, forming this group of experts across many different fields of science.

##### Hazlin Hazrin-Chong (Universiti Kebangsaan, Kuala Lumpur, Malaysia)

In 2016, Dr. Klas Udekwu gave a talk on MetaSUB research at a metagenomics conference in Malaysia. Being a microbiologist, I was intrigued by their work and asked Dr. Christopher Mason if Kuala Lumpur could join the effort. Since then, being part of this consortium has opened doors to discovering many otherwise hidden urban microbiomes in the world’s cities. This is particularly true in developing countries, where funding for such efforts is limited.

##### Paweł P. Łabaj (MCB Jagiellonian University, Kraków, Poland)

In 2015, Chris and I were at a dinner for the ISMB/ECCB2015 conference in Guinness Brewery. That was where he asked whether I would like to join a new consortium focused on scaling the work in his *Cell Systems* paper internationally. This is how I became a MetaSUB co-PI in Vienna and later in Kraków.

##### Olayinka O. Osuolale (Elizade University, Ilara-Mokin, Nigeria)

I also learned about MetaSUB through the MetaSUB website (www.metasub.org). Out of curiosity, I was browsing the latest in genomics and metagenomics aligning with my interests, and I came across the exciting metagenome papers and MetaSUB consortium. In 2016, I reached out to Chris to represent the cities in Nigeria, and since then, I haven’t looked back.

##### Christelle Desnues (Aix-Marseille Université, Centre National de la Recherche Scientifique, Marseille, France)

Our research interests are understanding the composition, diversity, and role of the viruses that surround and compose us. We were involved in the very early beginnings of the MetaSUB consortium, thanks to Nicolás Rascovan, a post-doctoral researcher in my group, who contacted Christopher six years ago. Since then, we are proud and lucky that the journey continues.

##### Ugur Sezerman (Acibadem University, Istanbul, Turkey)

I have been following Chris’s work since two of my students had started working with his group. We have been involved in several projects with MetaSUB in Turkey, including global City Sampling Day and MetaSEW. Besides good science, the real benefit is the companionship and the zest for science that the people involved in MetaSUB have. Everyone freely shares protocols, data, and supports each other’s research. We have so many collaborative projects going on within the consortium and we look forward to continuing to work with MetaSUB.

##### Alina Frolova (Institute of Molecular Biology and Genetics, Kyiv, Ukraine)

I was attending computational genomics school in Berlin as a student back in 2016, where Chris was an invited speaker. His talk was completely mind blowing as it was hard to imagine one person can do all that amazing science from space research to cancer genomics. He also mentioned MetaSUB and casually asked everyone to join. Now I think it was one of the best scientific decisions in my life to stalk him after the lecture as it resulted in a very fruitful collaboration and I’m super happy Ukraine is represented in MetaSUB.

##### Ding He (University of Copenhagen, Copenhagen, Denmark)

I joined the MetaSUB in 2019. The story started with me searching relevant metagenomics research protocols and resources when I was about to start my Marie Curie Fellowship at the University of Copenhagen. I came across MetaSUB’s website and found it very interesting. I started to explore MetaSUB’s mission and achievements, then realized that Copenhagen didn’t involve! I therefore started making contacts both to MetaSUB and to our local researchers. Copenhagen began participating gCSD in 2020, and we will continue to contribute to MetaSUB and sampling in 2022!

##### Julie Koch Sheard(University of Copenhagen, Copenhagen, Denmark)

I had been working with citizen science within the field of ecology for two years when Ding He asked me if I would help bring MetaSUB to Denmark. I was immediately excited by the thought, because the project differed from other citizen science projects, which typically involve recording large species observations. MetaSUB is a great project with strong opportunities for public engagement about metagenomics, a topic that might be foreign to many. It’s been fun talking to people on the subway about the MetaSUB project. These global scale studies are both timely and important.

### Around the world in one day: Coordinating “global city sampling day”

#### Did you encounter any challenges or any benefits of working with people from different backgrounds and expertise?

##### Mason

There were a number of challenges early on. During our initial sample collections, there were some variations in protocols (i.e., different swabs and reagents), and in order to have enough materials, we used different suppliers. It turns out sourcing sampling material for a global effort on a single day every year is not a minor undertaking.

We had to streamline the entire process for ambient sample collection methods, sequencing protocols, and analysis pipeline. It took a while to standardize everything and define the metadata to collect. For the past several years, however, the consortium has had a well-standardized protocol that ensures the use of identical swab collection protocol and metadata analysis pipeline, using methods from the Genome Standards Consortium. In fact, our coordination has smoothed to the extent that we now ship all the reagents and kits from New York City for global City Sampling Day (gCSD), which happens annually on June 21st, to all cities internationally.

##### Haruo Suzuki (Keio University, Tokyo, Japan)

We found that the metagenomic data obtained using different protocols were not always directly comparable (like apples and oranges). It is not possible to determine whether differences between metagenomic samples collected in different cities or years, and processed in different laboratories, are due to biological differences or protocol differences (so-called “batch effects”). To reduce any batch effects, the MetaSUB consortium members optimized and standardized all the protocols, and all the MetaSUB consortium sample processing was done at the same facility. http://metasub.org/methods/dna-extraction/.

##### Christos A. Ouzounis (AUTH/CERTH, Thessaloniki, Greece)

The coordinated effort in conducting and participating in gCSD is astounding. We have been trying for years with local authorities and academic colleagues to explore the power of metagenomics in the urban landscape, with slow progress and little success, but the protocols implemented by MetaSUB suddenly made planet-scale metagenomics possible.

##### Sampath Rapuri (St. Mark’s School of Texas, Dallas, USA)

As a high school student, it seemed like a daunting task to lead the sampling initiative in my city as well as recruit volunteers and engage with other youth citizen scientists. After explaining the consortium’s goals and strategies to potential volunteers, I was surprisingly met with an outburst of support from my peers. And through our involvement with MetaSUB, we have had a lot of fun as well as learned about fields like microbiology and genomics from leading researchers around the world, setting us up for future careers in science. I look forward to continuing our involvement in the consortium.

##### Krista Ryon (Weill Cornell Medicine, New York City, USA)

Initially, we were interested in mass transit systems, but we quickly learned that transit systems could be many things depending on where you are in the world—ranging from buses, motorcycles, subway systems, to even helicopters in Antarctica. So, as MetaSUB’s membership expanded, so did the scope of sampling day. We have representation from Greenland, where some cities have less than 50 people. At the same time, we have representation from cities that are some of the biggest in the world (e.g., Shanghai, New York, and Tokyo) as well as small villages and towns.

##### Gregorio Iraola (Institut Pasteur Montevideo, Montevideo, Uruguay)

Interdisciplinarity was always at the core of MetaSUB, I could feel it from the first day. For example, when we learned that MetaSUB was studying the microbiome of subway transport systems, we thought: what a pity that in Montevideo we do not have subways to analyze. However, we immediately thought we could extrapolate what was generated by MetaSUB to study other systems where microbes move in the city. This resulted in the first analysis of the sewage system microbiome in Montevideo (Fresia et al., 2019), which motivated the creation of new projects within MetaSUB like MetaSEW. In 2019, I created the Latinbiota Consortium (http://latinbiota.net), a network of collaborators from 8 countries from Latin America that is aiming to understand the composition and variation of the human microbiome at the continental scale. In collaboration with the Wellcome Sanger Institute (United Kingdom), we have performed shotgun metagenomics on more than 600 fecal samples. Comparison with global datasets revealed a massive expansion of microbial biodiversity associated with the human body. The integration of this data with environmental metagenomes from MetaSUB will enable unprecedented comparisons of the ecological relationships between the human microbiome and its surrounding environment.

##### Manuela Oliveira (University of Porto, Porto, Portugal)

Every year is an adventure. At one point, I was asked if I was working with a forensic lab: people assumed I was collecting DNA or fingerprint samples related to a crime that had just been committed before we got there. Last year, we stopped at another station to collect samples, and despite being accompanied by the Director of the Environment, Quality, and Safety Office and a member of the Security staff, someone made off with all of our sampling material. You really never know what to expect.

##### Klas Udekwu (Stockholm University, Stockholm, Sweden)

One of the more interesting MetaSUB memories I recall is, at one point, a graduate student was detained at a city’s police headquarters overnight on sampling day.

##### Mason

She ended up swabbing the holding cell, and I think that tells you everything you need to know about our members’ dedication to the project. If you get detained, you should *still* get a sample.

##### Ryon

This story serves as a good reminder that we have to consider public engagement in many aspects of this project. When sampling, we encourage all the cities involved in MetaSUB to reach out to their local officials and educate the public on why we are sampling the transit systems.

##### Daniela Bezdan (University of Tübingen, Tübingen, Germany)

It has been interesting how the MetaSUB consortium has evolved in the last five years. Due to public misconception and administrative challenges, there was initial resistance to global sampling. However, due to the increasing positive press coverage recently, the reception of those microbial studies in public is changing. Besides the scientific aspect, the educational aspect is also important to us, and we have seen an impact here.

##### Hazrin-Chong

Convincing the authorities and railway companies to grant us sampling permits was complicated, which I learned to be the norm for other cities with privately owned transport systems. The most challenging task in expanding globally, I think, is to have the public and authorities on board with what MetaSUB is doing, and how the scientific data derived from this research can benefit the public. Granted, COVID-19 has led to people/organizations being more interested in knowing what is in their local environment (e.g. sewage and hospitals). However, it is still a long road to change unwarranted, negative perceptions about microorganisms.

##### Eran Elhaik (Lund University, Lund, Sweden)

To get at this, we published a paper to address concerns and confusion about metagenomic efforts (Shamarina et al., 2017). Having an international Consortium and pursuing such orchestrated, rigorous scientific thinking has helped immensely in terms of communication. MetaSUB projects extend to schools, kindergartens, playgrounds, and other places. Everything is swabbed and studied, which alleviates the negativity prevailing about microbes.

##### Udekwu

The Stockholm regional government was extremely enthusiastic about the MetaSUB project, funding extensively our initial collections between 2016 and 2019. However, like Eran, I have experienced perplexed or at times adverse reactions. For example, we picked up SARS-CoV-2 in the Stockholm subway; as a result, sampling has not been allowed there since in order to avoid public concern. This is somewhat backward logic, and it illustrates that we need MetaSUB to be even more present internationally. Together, we can engage and educate government officials and the public, whereas our voices may not be heard individually.

##### Tiasse (Biodidact, Los Alamos, New Mexico, USA)

On the other end of the spectrum, however, many of our interactions with the public were positive, perhaps because we live in a very “science-oriented” town, home to a national laboratory (nearly ⅕ individuals have a Ph.D). Before sampling in Los Alamos, our volunteers received training on how to properly sample surfaces and fill out their form. The grocery store and the gas station are where we got more attention.

Volunteers reported first getting an inquisitive: “What are you doing?”, then a somewhat excited “That’s cool!”.

Three of our volunteers were homeschooling parents who took this opportunity to educate their children about the microbial diversity present in our environment. One of our volunteers chose to swab high-touch areas at our local police station. The officers welcomed the experience and were actually very excited to learn about MetaSUB and swabbing. Moreover, now, one of our high school student volunteers started working in our lab after the experience they had swabbing at Los Alamos.

##### Mason

In addition to navigating the complex public and government perceptions, we faced enormous logistical challenges as MetaSUB continued to expand. The projects really required tailoring collection protocols that worked very well anywhere, in marshes, polar regions, or other peculiar environments. For example, the collaboration with the CRG in Spain resulted in an improved and optimized protocol for high-throughput sampling. Even the app we use for data collection needed adjustment to work offline and online, e.g., in the tunnel and subway transit systems.

##### Ryon

What helped us overcome some of the most significant challenges is that we successfully established philanthropic and corporate partnerships, which are integral to enabling this science. The Alfred P. Sloan foundation first expressed interest in expanding our work beyond New York, helping us kick the entire project off. The Bill and Melinda Gates Foundation funding was also a big help, which led to other funding and recent support from the Rockefeller Foundation. Illumina donated some flow cells and other reagents, and Promega, New England Biolabs, and QIAGEN donated or discounted reagents and support. Arbor Biotechnologies helped with computational analysis and our annual meetings. We also received donations of DNA/RNA shield buffer to transport samples from Zymo Research, and they have also helped with centralized extraction and uniform processing methods. Informatics support from Amazon and Twist Bioscience donation of controls also helped a lot. These supporters are a demonstration of strong belief in the projects that we are engaged in.

##### Emmanuel Dias-Neto (A. C. Camargo Cancer Center, Sao Paulo, Brazil)

In 2010, we decided to revisit some data from our Human Cancer Genome Project (sponsored by the Ludwig Institute for Cancer Research and FAPESP, Brazil), which showed the presence of bacterial DNA in human tumors (Dias Neto et al., 2000). We needed approaches to evaluate the microbiota in large-scale and the progress was hindered by the consensus on sample collection, standardization of sequencing protocol, and many other logistical challenges for this emerging idea. After association with MetaSUB, though, we have largely resolved these issues. We continue to improve logistics, solve challenges, and track down microbial mysteries, but the fantastic group of people in MetaSUB discusses these problems openly to find a solution. For a consortium that is so diverse, we work well together.

##### María Mercedes Zambrano (CorpoGen, Bogotá, Colombia)

Under Chris’s leadership, the consortium does an incredible job at making people feel included, specifically in how his team has set up consortium-wide access to data, computational assistance, and reagent support. I am not a bioinformatician, but I learned a lot from observing the development of MetaSUB’s bioinformatic Core Analysis Pipeline (Danko and Mason, 2020). The experience and the scientific exchanges have been excellent. In fact, one of my laboratory-trained fellows is now a graduate student in Chris’ laboratory.

##### Łabaj

When I joined MetaSUB, my knowledge of metagenomics was limited, as I was coming from a background in transcriptomics. However, with every meeting, I realized that those two worlds are not that different. Of course, methods cannot be transferred directly, but it appeared that concepts and solutions might be easily adapted. A perfect example can be the work of our colleague Dr. Andre Kahles (ETH, Zurich, Switzerland). His concepts for graph-based sequencing data analysis took their origin in (cancer) transcriptomics. Over time, his team developed a much more powerful, generic framework for graph-based, large-scale metagenomics sequence analysis (https://metagraph.ethz.ch).

##### Andre Kahles (ETH, Zurich, Switzerland)

The MetaSUB project has been a huge catalyst for the development of the MetaGraph framework. Providing us with a very large, highly diverse dataset that was also annotated with detailed metadata, the project allowed us to develop and test the scalability of our approaches in a real-life context. As a result, we became pioneers in making all sequencing data of a project really full-text searchable. Another benefit is that since then many more use cases and research directions have emerged that will keep us busy with methods development for the coming years.

##### Le Huu Song and Thirumalaisamy P. Velavan (Vietnamese German Centre for Medical Research, Hanoi, Vietnam - Universitätsklinikum Tübingen, Germany)

Microbes are miraculous, they evolve much faster than humans, and this is also valid for MetaSUB. It has truly been evolving over the years.

Comparing microbial communities in different environments over time and space is very useful to understanding microbial dynamics, particularly in a hospital environment, which was our objective in Hanoi.

We began collaborating with MetaSUB, understanding the consortium as a global multidisciplinary team “united for purpose” in identifying microbes in different environments with a harmonized research design. With an exceptional project management team, MetaSUB has consistently delivered successful project outcomes in a timely and sustainable manner, sharing results with community and policymakers alike. All data generated by MetaSUB are open for anyone to view and analyze.

##### Frolova

At some point, I took an initiative to organize metadata collection, which means standardization of collected information (data types, which places to swab, input validation etc.) and choosing appropriate software solutions to work without internet (if needed), on any device and with minimum risks of data loss. On the other hand, the collection process should be flexible enough, because there is no single identical transport system in the world and we had to take into account local characteristics. However, later I realized the most difficult thing was to keep everything concise. It was my first global City Sampling Day when we designed a new collection web form, which looked perfect to me and we heavily tested it before the 21st of June. However, when I personally had to input over 100 samples at the end of the day, I was completely exhausted. Field work made me rethink our design. In general, I love this aspect about MetaSUB, despite futuristic goals we stay very practical. Luckily, that day we finished swabbing on time, because we had an amazing team of volunteers in Kyiv supported by Kyiv metro officials. And later thanks to Ben Young (Executive Director of MetaSUB, 2019–2021) and Krista Ryon (Executive Director of MetaSUB, 2021), we were able to greatly improve our sample collection process.

##### Zehra Hazal Sezer (University of Tübingen, Tübingen, Germany)

During my MA, while studying metagenome analysis, I also had the chance to present at the 2019 MetaSUB International Conference in Istanbul, Turkey. This was my first presentation at a conference and led to the opportunity to join the global City Sampling Day and MetaMED project. As a project member, I have attended meetings and colloquia, and been involved via research on COVID-19 in sample collection from one of the biggest hospitals in Istanbul. After gaining my MA, it was through the MetaSUB project that I found my current position in Tübingen, Germany. My involvement with the MetaSUB project allowed me to perform interdisciplinary research, gain experience in project management, and the chance to collaborate with inspiring scientists from around the world.

##### Afshin

Zehra’s experience is one of so many and is really one of the most significant accomplishments of MetaSUB: providing opportunities for individuals to grow, thrive, and launch their careers. When this all began, I was an undergraduate summer student working in Chris’ lab, and now I have my MD and bring these skills to the clinic. MetaSUB has played a critical role as I’ve started my career as a physician-scientist, and it has been amazing to see the scores of students (high school through graduate school), post-docs, and early-career professors get their start. In NYC alone, our team of swabbers, the “Swab Squad”, are largely made up of undergraduate students who volunteer on global City Sampling Day and they have gone on to pursue graduate school, medical school, and one has even become our current executive director!

### Beyond cities: Wastewater, hospitals, and monuments

#### What other types of samples does the consortium work with?

##### Mason

Because of the MetaSUB consortium and the collective effort of all the people associated with it, we now have a well-established infrastructure for both sampling and computational work. We currently have several spinoff projects. For example, we have initiated the MetaMED project to analyze samples from hospitals and medical environments. We also have Monumentome project, where samples are collected from monuments and landmarks, which can detail the causes of their degradation. We have already collected samples from 21 monuments and landmarks from 12 different cities, including surface samples from the ancient cities of Tel Megiddo in northern Israel, which follows some of the protocols we developed when doing metagenomics on the Dead Sea Scrolls (Anava et al., 2020). Some of our methods for k-mer mapping are now also used in projects with NASA for determining levels of possible biological contamination for spacecraft (Danko et al., 2021b).

The first significant MetaSUB spinoff project was the MetaAIR initiative launched in 2016. Dr. Marius Dybwad spearheads the MetaAIR initiative in Oslo and Dr. Patrick Lee in Hong Kong. MetaAIR is still going strong and has been running yearly air sampling campaigns synchronized with MetaSUB’s global City Sampling Day (gCSD) since 2017. The first MetaAIR results were published in *Microbiome* (Leung et al., 2021) on the same day the main MetaSUB paper was published in *Cell*.

We also have an ongoing MetaSEW project where we are sampling wastewater from 14 different cities with an added focus on COVID-19 surveillance and variant testing. In addition, we have a MetaCoV project from 27 different cities to characterize the changes in the urban metagenome to understand the presence of the SARS-CoV-2 virus. Interestingly, the Rockefeller Foundation’s Pandemic Prevention Institute (USA) is encouraging us to undertake a project for quantitative measurement of the environmental microbiome to predict infectious diseases and we are now working very closely with them. More details about these ongoing and future MetaSUB projects can be found at http://metasub.org/projects/, https://pngb.io/metasub-maps, http://extrememicrobiome.org and https://dnaloc.ethz.ch database.

##### Bezdan

One of my favorite MetaSUB spinoff projects is StuckOnU (http://metasub.org/stuckonu/), where we sequenced about 1,200 cellphones across six different conferences and quantified their microbiota.

Given that individuals bring their smartphones to all occasions and touch them frequently in all situations, questions arise regarding the classification, persistence, migration, and dynamics of bacteria and viruses on such personalized devices. Since we collected an extensive amount of metadata, such as geolocation, eating habits, and antibiotic use, we were able to associate abundance and living habits in a novel and exciting way.

##### Silvia Borras and Sofya Pozdniakova (AIRLAB–ISGlobal, Barcelona, Spain)

Our projects aim to disentangle the urban air microbiome and resistome, and its effects on human health. We are eager to join the next global City Sampling Day and the MetAIR initiative to expand the MetaSUB frontiers and continue the collaboration initiated by our colleagues at CRG in Barcelona.

##### Suzuki

We also have a project that aims to assess the impact of international movement of people and microbes as well as seasonal variation. We have swabbed the same selected locations/surfaces as Summer (gCSD2019 to gCSD2021) projects in Fall and/or Winter in over 20 cities in Japan. The time frames overlap the Rugby World Cup 2019 and the Olympic and Paralympic Games in Tokyo 2020. http://metasub.org/projects/the-olympiome-project/.

##### Malay Bhattacharyya (Indian Statistical Institute, Kolkata, India)

The analyses of the MetaSUB data have also evolved through an interesting open science approach. MetaSUB had partnered with the CAMDA (Critical Assessment of Massive Data Analysis) conference to promote the analysis of MetaSUB data through an open data challenge. This started in 2017 with “The MetaSUB Inter-City Challenge” followed by “The MetaSUB Forensics Challenge” in 2018 and 2019. This has generated a community of analysts who are continuously mining the MetaSUB data.

##### George Grills (University of Miami, Miami, USA)

What we are doing in many ways is we are creating dynamic maps of microbiome prevalence that are integrated with human data. The infrastructure that we are building and the approaches we are developing will allow us to create weather maps of microbiomes that could help predict where there might be surges of infectious diseases, e.g., COVID-19. Such predictions will then help put mitigation measures in place and devise a strategy for effective vaccine development.

##### Cynthia Gibas (University of North Carolina at Charlotte, Charlotte, USA)

We appreciate how joining the MetaSUB consortium adds value to our ongoing local wastewater surveillance program. We’ve been monitoring our university campus as a mitigation measure against COVID-19 outbreaks in the dorms, and we have archived over a year’s samples. Without immediate funding dedicated to metagenomic sequencing, the most we can do locally is to sequence the SARS-CoV-2 present in those samples. However, by contributing them to MetaSEW, we will have detailed information about the aggregate microbiome of our community across more than one season. In 2020, we collected surface samples in our public transit system vehicles to help the city determine whether sanitization measures were effective against COVID-19. We also sent samples to MetaSUB to better understand the microbial and viral material on those surfaces. In 2021, we were able to sample surfaces at the Charlotte airport, which is, I think, the first international airport to participate in gCSD.

##### Desnues

We are also now part of MetaSEW. We collected weekly wastewater samples from the second-largest city in France during the second and third waves of COVID-19. Joining this consortium was a real chance for science in our country, and we hope the collaboration will continue for the years to come.

##### Xavier Rodó (ICREA & ISGlobal, Barcelona, Spain)

We recently joined the consortium even though we were willing since I read Mason’s article in Cell. Since 2011, we have been sampling aerosols and realizing the exceptional biodiversity of microbes in the air. MetaSUB also has a MetaAIR component that we plan to expand to study how climate is contributing to the geographical differences we see in MetaSUB data.

##### Ouzounis

As Chris points out, MetaSUB was the launchpad project for many new, exciting activities. We are particularly thrilled with Monumentome, which has value for us as a community in a country with vast cultural heritage and unknown parameters about the preservation and restoration of ancient monuments and other artifacts. A new era for Archaeology in general and Conservation Science, in particular, is emerging with the study of microbiomes of cultural landmarks.

### Looking forward: The future of MetaSUB

#### Where do you see the future of MetaSUB?

##### Grills

Overall, what we are doing is a real example of a modern-day team science approach and bringing in colleagues with specialties in diverse fields. We have people from different backgrounds such as microbiology, ecology, bioinformatics, computational genomics, design, engineering, diagnostics, and clinical care. We have a highly miscellaneous array of specialties and expertise, working together very efficiently.

##### Tiasse

I second that. The way this team works together is quite impressive. Bringing together such a diverse group of scientists from different cultures and expertise while allowing respectful scientific discourse and still being productive is definitely a tour de force. I have to credit the team at Chris Mason’s lab for that. They do an excellent job, being inclusive in many ways. No contribution is undervalued. It’s a very collegial atmosphere.

##### Osuolale

I learned that once there is a unity of purpose, there is nothing impossible to achieve. Working with the consortium made me realize what it fully means to collaborate. Not just a word thrown around but everyone committing and contributing to the set tasks to achieving the set goals. Stepping down the MetaSUB activity in my cities of study made me learn the art of collaborating even with non-scientists, and also incorporating citizen science which engaged various individuals with different educated, semi-educated, and uneducated backgrounds. You never know what you will discover from an interdisciplinary study such as MetaSUB, I was super elated to see the kind of results that came out for the two cities (Offa and Ilorin) in Nigeria. It showed there are yet so many things undiscovered and not understood about our environment. This wouldn’t have been possible without the effort of the consortium. The resources, technicality, and the funding to execute such is practically impossible, so this interdisciplinary research lifted a bulk of the heavy weights in making the unique findings of our cities in Nigeria possible.

##### Łabaj

I think with our current momentum and level of collaboration, MetaSUB can head in some amazing directions. For example, a clear next step is to leverage our team into a global network of smart cities that can take advantage of microbial fingerprints. These can serve as baselines where any observed deviations from those could indicate an upcoming threat to our health and well-being. With the recent developments in (near) real-time metagenomic sequencing/detection and advancements in AI-based analytical and predictive models, we are at the edge of developing the Global AMR/Pathogen Spread Warning System. Moreover, I am convinced that the MetaSUB community will take the lead here.

##### Braden Tierney (Weill Cornell Medicine, New York, USA)

In addition to making groundbreaking biological discoveries, we are really well poised to add a lot by building cutting-edge bioinformatic tools. Building high-quality software that is easily used, easy to add to, and is managed collectively by a global consortium is extremely difficult. However, that is exactly what we are striving for. We are planning in the next few years to make the MetaSUB analytic tools the gold-standard for cutting-edge, bioinformatic analysis.

##### Hazrin-Chong

Apart from performing high-quality science, MetaSUB also has tremendous potential to increase the microbiology literacy of societies globally. Participation in the global city sampling day by members of the public is a great chance to educate them about microbes that live in, on, and around us. This heightened awareness and appreciation of microbes can potentially help mitigate future outbreaks and perhaps pandemics. Hopefully, the rise in public literacy on microbes would better support the science that MetaSUB and similar research consortia are doing in the long run.

##### Frolova

MetaSUB is growing bigger! I’m very excited to see the idea of the Global Pathogen Surveillance System become a reality. For better coordination between regional partners, we’ve established MetaSUB Europe society (https://metasub.eu/) currently chaired by Andre Kahles (ETH, Zurich, Switzerland), which will help us to involve even more stakeholders and spread the ideas of citizen science. Who knows, maybe MetaSUB Mars society is next?

##### Mason

We have so many ideas, projects, and, most of all, an incredible, international team all devoted to exploring microbiology together. There is so much to do, and I cannot wait to be part of growing the team in the years to come. The future of MetaSUB is very bright.
